# Expression and loss of alleles in cultured mouse embryonic fibroblasts and stem cells carrying allelic fluorescent protein genes

**DOI:** 10.1186/1471-2199-7-36

**Published:** 2006-10-16

**Authors:** Jon S Larson, Moying Yin, Jared M Fischer, Saundra L Stringer, James R Stringer

**Affiliations:** 1 Department of Molecular Genetics, Biochemistry and Microbiology, College of Medicine, University of Cincinnati, Cincinnati, OH 45267-0524, USA

## Abstract

**Background:**

Loss of heterozygosity (LOH) contributes to many cancers, but the rate at which these events occur in normal cells of the body is not clear. LOH would be detectable in diverse cell types in the body if this event were to confer an obvious cellular phenotype. Mice that carry two different fluorescent protein genes as alleles of a locus would seem to be a useful tool for addressing this issue because LOH would change a cell's phenotype from dichromatic to monochromatic. In addition, LOH caused by mitotic crossing over might be discernable in tissues because this event produces a pair of neighboring monochromatic cells that are different colors.

**Results:**

As a step in assessing the utility of this approach, we derived primary embryonic fibroblast populations and embryonic stem cell lines from mice that carried two different fluorescent protein genes as alleles at the chromosome 6 locus, ROSA26. Fluorescence activated cell sorting (FACS) showed that the vast majority of cells in each line expressed the two marker proteins at similar levels, and that populations exhibited expression noise similar to that seen in bacteria and yeast. Cells with a monochromatic phenotype were present at frequencies on the order of 10^-4 ^and appeared to be produced at a rate of approximately 10^-5 ^variant cells per mitosis. 45 of 45 stably monochromatic ES cell clones exhibited loss of the expected allele at the ROSA26 locus. More than half of these clones retained heterozygosity at a locus between ROSA26 and the centromere. Other clones exhibited LOH near the centromere, but were disomic for chromosome 6.

**Conclusion:**

Allelic fluorescent markers allowed LOH at the ROSA26 locus to be detected by FACS. LOH at this locus was usually not accompanied by LOH near the centromere, suggesting that mitotic recombination was the major cause of ROSA26 LOH. Dichromatic mouse embryonic cells provide a novel system for studying genetic/karyotypic stability and factors influencing expression from allelic genes. Similar approaches will allow these phenomena to be studied in tissues.

## Background

During malignant progression, cells accumulate multiple genetic and epigenetic alterations that cause loss of at least one anti-oncogenic function. Such a loss can be caused by a variety of events including mutation and losses that take place at the chromosome level, e.g. loss of heterozygosity (LOH), which is a hallmark of numerous cancers [1-5]. Many cases of LOH are caused by mitotic recombination (MR) between homologous chromosomes [6]. LOH can also arise via uniparental disomy (UPD), a change that presumably begins with nondisjunction of sister chromatids, producing trisomy in a daughter cell. Subsequent mis-segregation during mitosis of a trisomic cell can produce a disomic cell where both homologues were derived from the same parental homologue (UPD) [7]. On other occasions, gene conversion (GC) and interstitial deletions cause LOH [8,9]. In addition, it has recently come to light that some cells in the brain can be monosomic for one or more chromosomes [10].

Tumors serve as indicators of allele loss, but not all allele loss events necessarily lead to a tumor. LOH in diverse, nontransformed cell types in the body would be directly detectable if this event were to confer an obvious phenotype (other than tumorous growth) on an individual cell, its sibling, and their progeny. Mice that carry two different fluorescent protein genes as alleles of a locus would seem to be a useful tool for addressing this issue because LOH would change a cell's phenotype from dichromatic to monochromatic. If tissue architecture permits, the cause of LOH would be suggested by the number and arrangement of mutant cells because LOH caused by mitotic crossing over produces a pair of neighboring monochromatic cells expressing different colors [11]. By contrast, LOH caused by other events, such as UPD, gene conversion, or point mutation would be expected to produce a single monochromatic cell.

As a first step in assessing the utility of the allelic marker approach in mammals, we derived cell lines from mice that carried two different fluorescent protein (cyan and yellow) genes as alleles at the widely expressed ROSA26 locus, which is on chromosome 6 [12]. Although our studies were primarily motivated by an interest in LOH in mouse tissues, studies on genetic stability and allelic gene expression in mouse ES cells are of interest in their own right. The totipotent nature of ES cells has made them a useful tool for manipulating the genome and a promising prospect for human therapeutic applications. However, introduction of genetically damaged ES cells could lead to adverse outcomes.

The genetic and karyotypic stability of ES cells in general is not entirely clear. On one hand, aneuploid mouse ES cell lines are fairly common [13,14]. On the other hand, hundreds of mice have been made from ES cells, showing that these cells can maintain genetic stability when handled properly [15]. Some studies have suggested high rates of allele loss in ES cells [5,16], while in others, loss rates were hundreds of fold lower [17]. Different rates of point mutation have also been reported for ES cells [17,18]. The reasons for these different observations are not clear, but could include differences in marker genes employed, methods used to detect variant cells, cell lines studied, rates at which different chromosomes undergo either nondisjunction or mitotic recombination, and inadvertent selection of cells that proliferate better in culture. The bichromatic biallelic ES cells described herein differ from others studied because cells with variant phenotypes can be identified and isolated by FACS.

## Results

### Fluorescence phenotypes of cells in populations of biallelic embryonic fibroblasts

The two lines of transgenic mice used to make embryonic cell lines were a gift from F. Costantini, whose work had shown that both fluorescent proteins were simultaneously widely expressed in mice, which appeared normal [12]. Three R26CY mouse embryonic fibroblast (MEF) populations were independently derived, each from one 13.5 day post coitus (dpc) embryo. These cells were cultured for a few passages and then subjected to FACS analysis.

**Figure 1:**
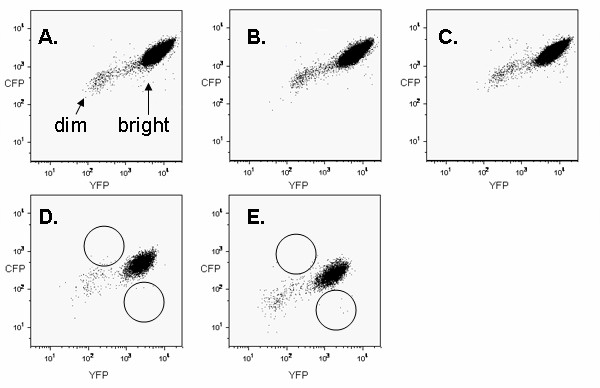
FACS dot plots (10^6 ^events) showing fluorescent profiles of three R26CY MEF cell lines (A-C) and two ES cell lines (D, E). Arrows in panel A indicate "dim" and "bright" events in MEF plots. Circles in panels D and E indicate areas where monochromatic variants would be expected to be located.

**Figure 2:**
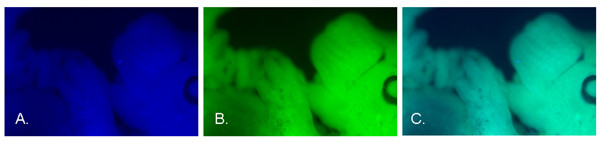
Expression of CFP (A) and YFP (B) in a mouse embryo (14.5 dpc), heterozygous at the ROSA26 locus. Panel C shows an image produced by overlaying the CFP and YFP signals.

Nearly all of the cells in each population exhibited both CFP and YFP fluorescence (bright cells) (Figures 1A–C). However, CFP signal intensities tended to be lower than YFP intensities, which was expected because CFP is intrinsically less bright [19]. A few percent of the cells exhibited little if any fluorescence of either color (dim cells). The nature of these cells was not investigated. Intact embryos appeared to express both fluorescent proteins uniformly and ubiquitously (Figure 2). Nevertheless, embryos could have contained a small number of cells that fail to express either fluorescent protein.

**Figure 3:**
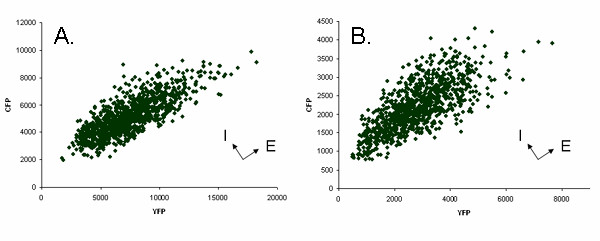
Variation in fluorescent signals (noise) from 1000 randomly selected cells. (A) MEF cell line 33e1 (B) ES cell line R26CY1. The fluorescent units are relative values assigned by the sorter. Intensities for CFP have been normalized to correct for its relative faintness when compared to YFP intensity. Arrows at right angles indicate noise axes, extrinsic (E) and intrinsic (I).

Figure 3A shows a scatter plot of YFP and CFP fluorescence intensities in 1000 individual MEFs drawn at random from the "bright" population shown in Figure 1. The points in this figure were plotted using CFP signal intensities that were normalized to correct for the inherent faintness of this protein. Different cells exhibited different levels of fluorescence and the fluorescence intensities varied over a 5 fold range on both axes. Such variation has been termed extrinsic expression noise [20,21]. However, in a given cell, YFP and normalized CFP signal intensities tended to be approximately equal. Coordinate variation in allelic expression has been observed in bacteria and yeast and is expected because the two alleles are exposed to the same intranuclear environment [20,21]. Nevertheless, lack of complete coordination of expression within a cell, a phenomenon known as intrinsic expression noise, is also expected, based on theory and on observations in *E. coli *and yeast [20,21]. MEFs that were brighter with respect to one color or the other can be seen as off-diagonal points in the scatter plot shown in Figures 1 and 3A. The intrinsic noise level exhibited by MEFs was approximately 0.2, a value similar to that reported for weakly transcribed loci in *E. coli *[20]. It was difficult to compare the MEF intrinsic noise level to those reported for yeast because yeast noise levels were measured for several different promoters under a variety of induction-repression conditions and reported in arbitrary units [21]. However, comparison of scatter plot shapes (i.e. the distribution of points relative to a diagonal line, compared to the range of fluorescence signals in the population) suggested that MEF and yeast intrinsic noise levels were generally comparable.

**Figure 4:**
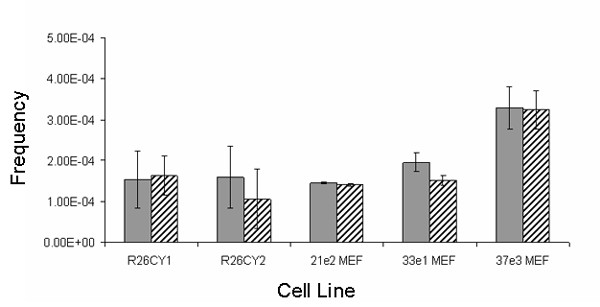
**Abundance of spontaneously occurring apparent monochromatic variants**. Solid and hatched bars indicate frequencies of cells exhibiting only CFP or only YFP, respectively. R26CY1 and R26CY2 are data from the two ES cells lines. 21e2MEF, 33e1MEF and 37e3MEF are data from the three MEF lines. Error bars indicate the standard errors of the means.

A few cells in each MEF population exhibited more than a 5 fold difference in CFP and YFP fluorescence and were suspected of being monochromatic (Figure 1). The number of cells expressing YFP only was approximately the same as the number of cells expressing CFP only (Figure 4). Apparent monochromatic cells of both colors were selected by sorting and placed in culture, but did not survive, precluding further phenotypic and genetic analysis. The reason for the failure of these cells to form clonal colonies was not investigated, but factors that might have caused this result include the low plating efficiency of single MEFs and the fact that the populations that were sorted had been passed several times prior to sorting.

### Fluorescence phenotypes of cells in populations of biallelic ES cells

Two R26CY ES cell lines were independently derived and studied. Figures 1D and 1E show FACS data obtained from these two cell lines. In addition to the points produced by ES cells, the FACS plots contained a relatively small number of points produced by autofluorescence of the feeder cells present in the ES cell cultures.

The fluorescent phenotypes in the two ES cell lines were similar to those seen in the three R26CY MEF cell lines (described above) except that the fluorescence emitted by each protein in a typical ES cell was about a third as intense as that seen in a typical R26CY MEF cell. Nearly all of the ES cells in each population exhibited both CFP and YFP fluorescence (Figures 3B). In a given cell, normalized CFP and YFP signal intensities tended to be approximately equal, but coordination of expression was not perfect. The two ES cell lines, R26CY2, exhibited intrinsic noise levels of 0.2, similar to that exhibited by R26CY MEFs.

Noise is expected to produce cells that exhibit CFP and YFP fluorescence intensities that differ over time. To determine if this were the case, a population in which the cells were two fold brighter with respect to CFP than YFP was obtained by FACS, placed in culture and passed 3 times. Analysis by FACS showed that the original phenotype (brighter CFP) was not maintained. Instead the population resembled those shown in Figure 3. Hence, the phenotype of the population was transient, as would be expected if it were due to noise.

**Figure 5:**
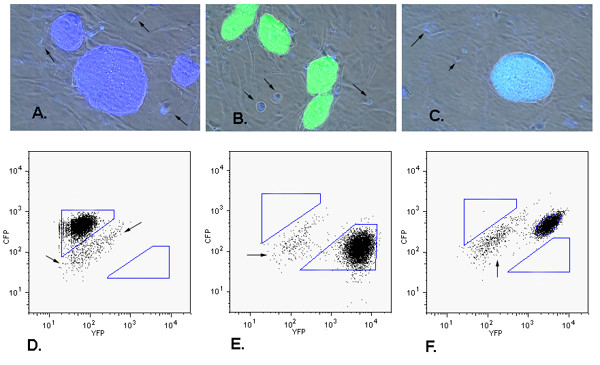
**Phenotypic analysis of FACS-isolated ES cell clones**. Panels A-C show images of three clones with different phenotypes. (A) colony of cells expressing CFP only, (B) colony of cells expressing YFP only, (C) colony of cells expressing both CFP and YFP. Images shown were are produced by merging three images, CFP epifluorescence, YFP epifluorescence, and phase contrast. Gray cells under the ES clones are a monolayer of non-fluorescent wild type MEF feeder cells. Arrows indicate some of the cellular debris and dead cells that exhibited autofluorescence. Panels D, E and F correlate with panels A, B and C, respectively, and show FACS analysis of each subclone. Polygons indicate gates used to collect putative monochromatic cells. Arrows indicate residual MEF feeder cells present in ES cell populations subjected to FACS.

Both R26CY lines contained rare cells that appeared to be monochromatic (Figure 1). These cells occurred at a frequency of approximately 10^-4 ^and apparent CFP and YFP monochromatic variants occurred in roughly equal numbers in both ES cell populations (Figure 4). To determine if they were stable variants, apparent monochromatic cells were gated into collection tubes, plated at low density and cloned. Examination of 85 clonal cultures by fluorescent microscopy showed that about 80% of them exhibited the expected monochromatic phenotype (Figure 5). The remaining 20% were dichromatic, indicating that the sorting method produced populations of cells that were highly enriched for monochromatic cells, but that some dichromatic cells passed through the gates. Subsequent experiments using mixtures of monochromatic and dichromatic ES cells showed that using more narrow gates reduced the recovery of true monochromatic cells (data not shown). Therefore, quantification of monochromatic cells was more accurate when gates were set wide and dichromatic cells that were misidentified by FACS were later detected by microscopy.

### Spontaneously arising stable monochromatic ES cells lacked the gene encoding the non-expressed fluorescent protein

**Figure 6:**
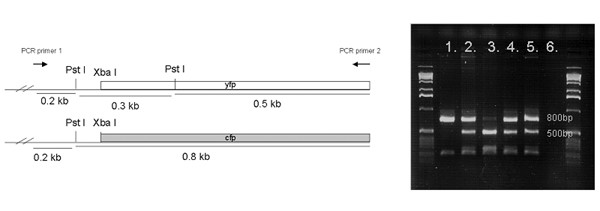
**Genotyping the ROSA26 locus in DNA isolated from monochromatic clones**. At left are shown maps of the two alleles in parental cells. The YFP gene has an additional cleavage site for Pst1 restriction endonuclease. Lane 1, a clone lacking the YFP gene. Lane 2, a dichromatic clone, which retained both the genes, as expected. Lane 3, a clone lacking the CFP gene. Lanes 4 & 5, data from DNA of unsorted ES cell lines. Lane 6, PCR control containing no DNA in the reaction mix. Markers are 1 Kb DNA ladder (Invitrogen).

To determine if gene loss contributed to the production of monochromatic ES cells, PCR was used to amplify fluorescent protein genes and amplicons were analyzed by digestion with the restriction endonuclease Pst1 because the YFP gene has a Pst1 cleavage site that the CFP gene lacks (Figure 6). All spontaneously occurring monochromatic ES cell clones analyzed by restriction enzyme analysis (n = 45) lacked the gene encoding the absent fluorescent protein. To confirm these results, PCR products from 7 monochromatic ES cell clones (3 expressing only YFP and 4 expressing only CFP) were cloned and sequenced. At least 5 cloned amplicon copies from each of the 7 monochromatic ES cell clones were sequenced. All of the sequences from a given monochromatic cell line were identical to the gene encoding the fluorescent protein observed in that cell line. As a control, PCR products from a dichromatic ES cell population were cloned and sequenced. Sequences from CFP and YFP genes were both present and equally abundant, as expected. Three additional monochromatic clones were analyzed by Southern blot hybridization, which showed that each had lost the gene encoding the absent fluorescent protein (data not shown).

### Analysis of a centromeric marker in cells with LOH at ROSA26

To investigate the nature of the events that produced allele loss at the ROSA26 locus, a centromeric heterozygous microsatellite (D6Mit159) was identified. The D6Mit159 locus is 30 Mbp from the centromere and 83 Mbp from the ROSA26 locus. Heterozygosity was retained at the D6Mit159 locus in 12 of 20 spontaneous monochromatic clones examined, suggesting that 60% of spontaneous monochromatic clones were produced by mitotic crossovers within the 83 Mbp interval between the D6Mit159 microsatellite marker and ROSA26. In the other 8 monochromatic clones, heterozygosity was lost at the D6Mit159 locus. Such a result was consistent with loss of one homologue of chromosome 6, although LOH at both D6Mit159 and ROSA26 might have been caused by mitotic recombination taking place in the 30 Mb interval between the centromere and D6Mit159.

**Figure 7:**
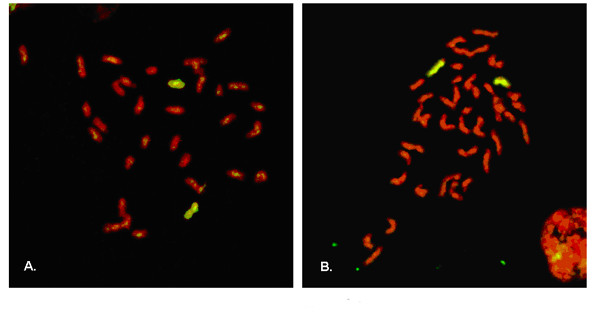
**Whole chromosome-6-paint FISH analysis of monochromatic ES cell clones that had lost heterozygosity at both the ROSA26 locus and D6Mit159**. Metaphase chromosomes were hybridized to a chromosome 6 probe labeled with FITC. Chromosomes were counter stained red with propidium iodide (PI). Images shown were produced by merging FITC and PI epifluorescence images. Copies of chromosome 6 are yellow. Other chromosomes are red. Panels A and B show metaphase chromosomes from cells that expressed CFP only or YFP only, respectively. 400× magnification.

The possibility of chromosome 6 monosomy was tested by whole chromosome painting of metaphase chromosomes in two of the 8 monochromatic clones that were homozygous at D6Mit159. All metaphase spreads examined were disomic for chromosome 6 (Figure 7). These data, along with the fact that autosomal monosomy has not been described in mouse ES cell lines, suggested that chromosome loss without re-duplication was not a major contributing mechanism of allele loss.

### Copy number of chromosome 6 in parental ES cell lines

UPD would explain the monochromatic cells that were disomic for chromosome 6 yet had LOH near the centromere. Development of UPD would be facilitated by trisomy for chromosome 6 in the parental cell lines. Therefore, it was of interest to determine if either of the parental R26CY ES cell lines had this karyotype. To that end, 66 cells in cell line R26CY1 were subjected to spectral karyotyping, and 42 cells in cell line R26CY2 were analyzed by whole chromosome painting. All cells analyzed contained 2 copies of chromosome 6. These data established with 95% confidence that the fraction of cells with trisomy 6 was 5% or less in cell line R26CY1 and 7% or less in cell line R26CY2.

### Frequency of monochromatic ES cells and estimated rate of LOH

The original FACS experiments showed that monochromatic variants occurred at frequencies of 3.1 × 10^-4 ^and 2.6 × 10^-4 ^in the R26CY1 and R26CY2 ES cell lines, respectively. These data were acquired from populations of cells that had been derived from blastocysts and kept in culture for several months. Thus, it was possible that the frequencies of monochromatic variants were inflated by their accumulation over time. To examine the relationship between frequencies of monochromatic cells and the rate at which they arise, preexisting monochromatic cells were removed from populations of R26CY cells by FACS. These dichromatic cell populations were expanded for 10 population doublings in culture, and then subjected to FACS analysis. Monochromatic cells were present on the order of 10^-4 ^in both cell lines. Monochromatic cells expressing only blue were approximately as frequent as those expressing only yellow.

Variants can be either more of less frequent than otherwise dictated by their rate of formation if they proliferate more or less rapidly than parental cells. To determine if monochromatic cells might proliferate more rapidly than their dichromatic parents, growth kinetics of clonal populations of different fluorescent phenotypes were studied. The different clonally derived populations exhibited a variety of growth rates, but fluorescent phenotype and growth rate were not correlated (data not shown). In addition, an increase in proliferation rate upon loss of one fluorescent protein seems an improbable scenario for two reasons. First, monochromatic cells did not exhibit less fluorescent signal than dichromatic cells, suggesting that the amount of fluorescent protein in monochromatic cells was not less than in dichromatic cells. Second, although it has been reported that it is possible to cause ill effects by over expressing GFP [22], there is little evidence of general toxicity associated with fluorescent proteins, which have been used in many ES cell lines [23-27] and in transgenic mice, which are often generated from ES cells expressing one fluorescent protein or another [12,24-29]. In the case of mice carrying CFP and YFP genes at ROSA26, the animals are viable and reproduce normally, as do mice homozygous for either CFP or YFP at ROSA26 [12].

These data suggested that the frequencies of monochromatic cells present in populations of R26CY cells reflected the rate of their production and that the two types of monochromatic variants arose at essentially the same rate, which can be estimated from the relationship between frequency of variants and the number of reproductive cycles (generations) the population has undergone, where rate equals the proportion of variants in a final culture divided by the number of generations that have elapsed [30,31]. By this calculation, the rate of LOH in both cell lines was approximately 10^-5 ^per cell-generation.

### Induction of mutation in bichromatic ES cells

Because all 45 spontaneous monochromatic ES cell clones examined exhibited LOH at the ROSA26 locus, it was of interest to determine if LOH were the only pathway capable of producing this phenotype. Therefore, R26CY1 ES cells were treated with ethylmethanesulfonate (EMS), which is a strong inducer of point mutations. The cell population exposed to EMS exhibited 1.7 fold more monochromatic cells as assessed by FACS followed by microscopy.

Twenty-five monochromatic clones isolated from EMS-treated R26CY1 ES cells populations were subjected to DNA analysis by PCR followed by Pst1 digestion. Five of the 25 clones retained both the CFP and YFP genes, suggesting that a point mutation had caused loss of expression of one fluorescent protein gene in 20% of the monochromatic cells produced following treatment with EMS. To test this hypothesis and determine the nature of these mutations, 3 of the clones that retained both fluorescent protein genes were subjected to sequence analysis. As expected, all 3 monochromatic clones harbored a mutant version of the non-expressed fluorescent protein gene, and the mutation predicted an alteration in the encoded protein sequence (Table 1). The mutation found in clone R26CY 1025 CFP-8, which expressed CFP but not YFP, explained the lack of YFP fluorescence because the YFP gene contained a frameshift mutation predicted to completely block production of the YFP peptide by stopping translation at codon 4. The mutations observed in the other two biallelic but monochromatic ES cell clones altered the amino acid sequence and were predicted to change the protein in ways that could extinguish fluorescence.

## Discussion

Populations of biallelic dichromatic mouse embryo cells contained numerous cells in which fluorescence of CFP and YFP were not equivalent. In about one in ten thousand cells, CFP and YFP fluorescence were highly disproportionate. This phenotype arose by the spontaneous loss of the gene encoding either CFP or YFP. The number of cells with this type of LOH could be determined by a combination of FACS followed by microscopic observation of populations originating from individual sorted cells.

While rare mutant cells exhibited highly disproportionate fluorescence, similar but nonequivalent CFP and YFP fluorescence was seen in most of the cells in biallelic dichromatic populations. These variations in relative fluorescence were transient and presumably produced by expression noise.

### Genetic instability in mouse ES cells

The rate of LOH inferred from the frequency of monochromatic ES cells is similar to rates reported in most other studies on LOH in mouse ES cells. LOH rates at 11 different mapped loci each carrying an inserted *neo *gene have been reported to range between 10^-3 ^and 10^-5 ^events per cell generation [5,16,32]. Experiments on cells carrying neo genes inserted at unknown loci produced similar results [33]. Studies using other markers also reported rates of LOH within the range suggested by the frequency of monochromatic cells [34,35]. In contrast to the rates reported in most studies, experiments using ES cells heterozygous at the *Aprt *locus indicated that spontaneous LOH occurred at a rate of approximately 1 × 10^-7 ^events per cell generation [17]. It is not clear why the rate of allele loss at the *Aprt *locus differed from those seen at other loci, but it is possible that chromosome 8 behaves differently from other chromosomes in this respect.

In mouse ES cells heterozygous at the *Aprt *locus, about 40% of LOH events had occurred via mitotic recombination and UPD was the most common genetic change associated with LOH at *Aprt *[17]. Similar results were reported for the *FasI *locus, where a third of the cells with LOH were produced by mitotic recombination [32]. Lefevbre et al showed that LOH with respect to integrated *neo *genes was accompanied by LOH at linked markers, but did not attempt to distinguish between mitotic recombination and UPD as the cause of these events [5].

Our studies on biallelic dichromatic mouse ES cells suggest that LOH at ROSA26 occurred principally by mitotic recombination occurring between ROSA26 and the DMit159 marker that is 30 cM from the centromere. However, because a heterozygous locus telomeric to ROSA26 could not be found, it is not possible to exclude interstitial deletion encompassing the ROSA26 locus but too small to produce an obvious decrease in the size of chromosome 6. Nevertheless, interstitial deletion seems an unlikely contributor because such events rarely generate spontaneous LOH in ES cells [6,36-39].

About 40% of the cells had LOH at both D6Mit159 and ROSA26. This genotype might have been caused by mitotic recombination taking place in the 30 Mb interval between the centromere and D6Mit159. However, the frequency of monochromatic clones with LOH at both D6Mit159 and ROSA26 was two fold higher than would be expected to be produced solely by mitotic recombination, assuming these events occur in proportion to the distance between markers. We would expect recombination in the 30 Mbp interval between the centromere and the D6Mit159 locus to occur 36% (30/83) as frequently as recombination in the 83 Mbp interval between the D6Mit159 locus, where recombination produced 12/20 (60%) LOH events. Therefore, only 4 of the 8 clones with LOH at D6Mit159 would seem to be attributable to mitotic recombination. Mechanisms other than mitotic recombination that might have contributed to the development of LOH at both loci include UPD and monosomy. UPD seems more likely because monosomy is very rarely seen in mammalian cells and has not been seen at all in mouse ES cells [36,39].

### Potential of biallelic fluorescent markers for studies on genetic instability

Findings obtained in these studies on biallelic dichromatic ES cells suggest that this approach can be extended to tissues isolated from mice. GFP has been shown to be useful for detecting chromosome loss in Hela cells and in mouse brain [10,40]. In a similar fashion, monochromatic cells in tissues from dichromatic mice can be identified and isolated by FACS. In addition, it may be possible to detect monochromatic cells *in situ *in tissue sections. While other approaches provide data on mutation and mis-segregation events in tissues, only the dichromatic model can provide information about the locations of variant cells within tissues, and this information has the potential to reveal mitotic recombination events because such events can generate twin spots composed of neighboring patches of monochromatic cells of different colors descended from the monochromatic daughter cells produced from a mitotic cell that has undergone crossing over between homologous chromosomes [11].

### Sources and features of expression noise

Mouse cells exhibited both extrinsic and intrinsic noise, the two types of variation seen in expression of allelic genes in yeast and bacteria [20,21]. Extrinsic noise refers to variation in the fluorescence intensity emitted by a given fluorescent protein in different cells in the population [20,21], and is thought to be due to variation among cells with respect to parameters such as position in the cell cycle. ES cells and MEFs exhibited similar levels of extrinsic noise, which may seem surprising given that MEF populations are derived from the numerous cell types present in a 13.5 dpc embryo, while ES cell populations contain a single cell type. However, many of the diverse cell types present in a dissociated embryo appear to fail to proliferate, and cells resembling fibroblasts quickly predominate in MEF cultures. Another factor that may work to minimize extrinsic noise caused by heterogeneity with respect to cell types is the robust activity of the ROSA26 promoter, which is driving transcription of the fluorescent protein genes. This promoter is known to function in a wide array of cell types [41-50].

Intrinsic noise refers to the discordance in the intensities of the two fluorescent proteins within a single cell, and is thought to be due to lack of coordination with respect to processes such as assembly of transcription complexes at allelic promoters [20,21]. This lack of coordination is thought to result from stochastic variation caused by a scarcity of factors needed to accomplish gene expression. Intrinsic noise occurred in both types of mouse cells, and to similar extents. The level of intrinsic noise exhibited by mouse cells resembled that seen in *E. coli *when the promoter driving fluorescent gene transcription was semi-repressed by the lac repressor protein [20]. Intrinsic noise in *E. coli *was reduced when repression was lifted and transcription rate was increased, leading to the suggestion that intrinsic noise is inversely related to transcription rate [20]. However, studies in yeast showed that the relationship between intrinsic noise and transcription can be more complex [21].

## Conclusion

LOH at ROSA26 produced monochromatic cells in populations of dichromatic mouse embryonic cells and was usually accompanied by retention of heterozygosity at a locus between ROSA26 and the centromere, suggesting that mitotic recombination was the major cause of ROSA26 LOH. Dichromatic mouse embryonic cells exhibited expression noise, a phenomenon previously described in bacteria and yeast carrying different fluorescent protein genes as allelic markers. Dichromatic mouse embryonic cells provide a novel system for studying genetic/karyotypic stability and factors influencing expression from allelic genes in cultured ES cells, and suggest that similar approaches will allow these phenomena to be studied in tissues.

## Methods

### Mice

The two lines of transgenic mice used to make embryonic cell lines were a gift from F. Costantini [12]. One transgenic mouse line (R26R-EYFP) carried a gene encoding enhanced YFP at the ROSA26 locus. The other mouse line (R26R-ECFP) carried a gene encoding enhanced CFP at the ROSA26 locus. We crossed the two lines to produce mice with different fluorescent protein markers at the ROSA26 locus. These mice were of mixed genetic background that included alleles from three inbred strains, 129X1/SvJ, C57BL/6J and FVB/n. This situation was due to the following history of mouse production and maintenance. Strain 129X1/SvJ was the background into which the fluorescent protein genes were originally integrated into the mouse genome by gene targeting [12]. The targeted 129X1/SvJ ES cells were injected into C57BL/6J blastocysts and chimeric mice were bred to C57BL/6J females to obtain transgenic mice that were 129X1/SvJ/C57BL/6J hybrids. The fraction of alleles from the 129X1/SvJ background was reduced from 50% because the mouse lines were maintained by crossing to C57BL/6J mice. The degree of this reduction can only be estimated because the number of crosses that had been performed since derivation of the original 129X1/SvJ/C57BL/6J hybrid transgenic lines was not available. The FVB/n background was introduced when the R26R-ECFP and R26R-EYFP mice were each crossed to a line of FVB/n mice that expresses the Cre recombinase during early embryonic development [12]. This cross was necessary to remove a floxed transcriptional stop cassette situated at the beginning of each fluorescent protein gene. We screened the offspring of these crosses and found that some of the mice expressed fluorescent protein ubiquitously, and analysis (by PCR) of the transgenes in fluorescent mice showed that the stop cassette had been removed. These results were as expected based on previous reports [12]. We selected one mouse exhibiting YFP fluorescence and one exhibiting CFP fluorescent and used them to establish two lines (R26YFP and R26CFP) by inbreeding. Hence, it was expected that most loci in the R26YFP and R26CFP lines would be occupied by alleles from either C57BL/6J or FVB/n, although alleles from strain 129X1/SvJ could be present at some loci.

### Derivation, culture and treatment of cell lines

Mouse embryonic fibroblast (MEF) polyclonal cell lines were derived as described [51]. Briefly, R26YFP and R26CFP mice were mated and at 13.5 dpc pregnant mice were sacrificed. Embryos were harvested and heart, liver and blood were removed and discarded. The remaining tissue was minced and pieces suspended in 1 ml 0.05% trypsin-EDTA (Invitrogen, Carlsbad, CA) and subjected to 12–18 hour digestion in at 4°C. Following digestion, cells were mechanically disaggregated by repeated pipetting in Dulbecco's modified eagle media (DMEM), supplemented with 10% FBS (Invitrogen, Carlsbad, CA) and 200 mM L-glutamine (Invitrogen, Carlsbad, CA) (complete DMEM). Cells from one embryo were divided among four 10 cm cell culture dishes and cultured overnight in DMEM, 200 mM L-glutamine, 10% FBS. These cells were trypsinized and cells divided among four plates. A day later, the cells were harvested and cryopreserved. For analysis, a vial of cells was thawed and placed in complete DMEM in a single 10 cm culture dish, which was incubated at 37°C until the plate was confluent (12 to 24 h).

To obtain ES cells, male R26YFP mice were crossed to female R26CFP mice. Derivation of ES cells was as described [51]. Briefly, blastocysts were harvested at embryonic day 3.5 and put into culture in ES cell media (Dulbecco's modified eagle media (DMEM) that was supplemented with 15% certified FBS (Invitrogen, Carlsbad, CA), 200 mM L-glutamine (Invitrogen), 0.1 mM β-mercaptoethanol (Sigma, St. Louis, MO) and leukemia inhibitory factor (LIF), 1000 U/ml (Chemicon, Temecula, CA)). Two cultured blastocysts each yielded a cell line that maintained morphology characteristic of mouse ES cells.

ES cell lines were maintained on primary embryonic mouse fibroblast (MEF) feeder layers, mitotically inactivated by treatment with mitomycin C (Fisher, Pittsburgh, PA) 0.01 μg/ml in DMEM for 1–2 hours, using standard incubation conditions: 37°C, 10% CO_2 _[51].

ES cells were mutagenized by the procedure described by Munroe et al. [18]. Briefly, approximately 1 million ES cells on a feeder layer were exposed to culture media containing 0.6 mg/ml ethylmethanesulfonate (EMS) for 20 hours, at which time culture media was removed, cells were washed several times and then placed in ES cell media and incubated at 37°C for 10 days. Cells were harvested and analyzed by FACS.

### FACS analysis

Cells were removed from plates by digestion with 0.25% trypsin-EDTA (Invitrogen, Carlsbad, CA) at 37°C for 5 to 15 min. Cells were suspended in sorting buffer (Ca^++ ^& Mg^++ ^free PBS, 1 mM EDTA, 25 mM Hepes,1%FBS), transferred into Falcon FACS tubes with cell strainers (35-2235)(BD Biosciences, San Jose, CA) and examined by light microscopy to verify the absence of aggregates.

Cells were sorted using a FACSVantage™ SE (BD Biosciences, San Jose, CA) flow cytometer equipped with digital DiVa Software, an argon laser (488 nm), Coherent Innova krypton laser tuned to 407 nm, 90 micron nozzle using a sheath pressure of 26 pounds per square inch, standard optics including a 530/30 nm band-pass filter (FL1) for YFP and 480/30 nm band-pass filter (FL4) for CFP. Daily alignment was performed using chicken red blood cells (BioSure, Grass Valley, CA) and AlignFlow Plus UV beads (Molecular Probes, Eugene, OR) for the 488 nm laser and UV laser, respectively.

Cells were first gated based on forward and side scatter. Then, cells were analyzed for both CFP and YFP fluorescence. Gates for isolation of monochromatic embryonic mouse cells were established using cells (either MEF or ES) made for the purpose. These cells carried and expressed either a YFP or a CFP gene. Sorted cells were collected into 1.5 ml conical tubes containing cell culture media, supplemented with 1× antibiotic-antimycotic (Invitrogen). Cells recovered from the FACS were cultured in media containing antibiotics.

ES cells subjected to FACS were harvested from plates that contained both transgenic ES cells and wild type MEF feeder cells. Although MEF feeder cells did not carry either fluorescent protein, it was expected that these cells might contribute to the FACS data due to autofluorescence. In fact, the FACS data produced by ES cell cultures contained a small number of faintly fluorescent points. To determine if these points were from feeder cells, the dim objects were isolated and placed in culture dishes. The next day, the dishes were inspected by microscopy, which showed the presence of adherent cells that were morphologically identical to feeders and were dimly fluorescent.

Data files were saved for every sort and subsequently analyzed using FlowJo software (TreeStar, San Carlos, CA). Frequencies reported are based on analysis of 2.5 × 10^6 ^events per sort. Statistical comparisons were performed using the Student's paired T-test.

### Noise calculations

Intrinsic and extrinsic expression noise values were calculated using formulas previously described [20,21]. Calculations were performed using CFP fluorescence values that had been adjusted to correct for the intrinsic faintness of CFP molecules compared to YFP molecules. An adjustment factor was determined for each cell line by comparing the population mean YFP fluorescence to the population mean CFP fluorescence. This factor varied slightly from one cell line to the other, but, generally, CFP fluorescence values used in noise calculation were approximately 2 fold higher the values reported by the FACS.

### Rate estimation

Rates of allele loss were obtained by studying the frequency of monochromatic variants in populations that arose during the expansion of populations that initially lacked monochromatic variants, which were obtained by FACS. Rates were estimated by dividing the proportions of variants by the number of generations estimated to have elapsed during expansion of initially variant-free populations [30,31].

### Phenotypic analysis of R26CY subclones

FACS-isolated subclones were expanded in culture and then analyzed by fluorescent microscopy on a Nikon E400 microscope. The filter set for CFP provided excitation light wavelengths between 426 and 446 nm and allowed detection of emitted light between 460 and 500 nm. The filter set for YFP provided excitation light wavelengths between 490 and 510 nm and allowed detection of emitted light between 520 and 550 nm. Experiments with monochromatic cells showed that the signals from the two fluorescent proteins could be detected without interference of one with the other. Images were captured using a Spot Jr. CCD camera (Diagnostic Instruments, Sterling Heights, MI).

### DNA analysis of R26CY subclones

ES cells were first separated from feeder cells by FACS. This separation was easily accomplished because feeder cells were not very fluorescent. DNA to be used for PCR was isolated from cells by proteinase K digestion in lysis buffer (5 mM EDTA, 200 mM NaCl, 100 mM Tris, 0.2% SDS) 8–12 hours at 57°C followed by isopropanol precipitation. PCR was performed in 50 μl containing 2.5 mM MgCl_2 _(Promega, Madison, WI), 1× PCR buffer (Promega), 1.6 mM dNTPs, 0.5 U Taq polymerase (Promega).

To analyze the ROSA26 locus, Primer 1 (5'CAGTAGTCCAGGGTTTCCTTGATG) which is specific to the ROSA26 promoter region, was paired with Primer 2 (5'GTCGCGGCCGCTTTACTTGT), which is common to both the CFP and YFP genes. Cycling conditions were as follows: 3 min denaturation at 94°C followed by 35 cycles of 94°C for 1 min; 58°C for 1 min, 72°C for 1 min, after which there was a final 5 min extension reaction at 72°C. The 1 Kb (approximate size) product was purified using the QIAquick PCR purification kit (Qiagen, Valencia, CA). CFP and YFP PCR products could be distinguished by restriction fragment length polymorphism (RFLP) analysis with Pst1 endonuclease (10 U/μl)(Invitrogen) using gel electrophoresis (1.5% agarose). The CFP product contains one Pst1 recognition site yielding bands of roughly 800 and 200 bp in size. The YFP product has two Pst1 sites and generates bands of approximately 500, 300 and 200 bp in size. To confirm the results of the Pst1 RFLP analysis, PCR products were cloned into TOPO-TA (Invitrogen) and sequenced. Statistical significance was tested using the binomial distribution.

LOH at ROSA26 might be accompanied by LOH at linked loci. To identify linked loci that would be informative, i.e. heterozygous in the parental ES cell lines R26CY1 and R26CY2, were sought. The first step was to identify candidate loci using the Mouse Genome Informatics (MGI) database [52]. Several loci were then tested by PCR and sequencing performed by Polymorphic DNA technologies (Alameda, CA) [53]. The D6MIT159 locus was found to be heterozygous. Genomic DNA samples were subjected to PCR using the primers described in the MGI database. PCR products were resolved using gel electrophoresis through a 3% agarose gel. Parental cells produced the two bands expected. Cells that had lost heterozygosity at D6MIT159 produced only one band. In all cases of LOH at D6MIT159, the band that was retained was the one linked to the ROSA26 allele retained.

### Cytogenetic analyses

Metaphase chromosome preparations for spectral karyotyping (SKy) analysis were prepared by treating 50–70% confluent cultures with 10 μg/ml colchicine (Sigma) for 2 hours. Following treatment, the cells were lifted with 0.25% trypsin, re-suspended in ES cell media, collected by centrifugation at 1300 RPM for 5 minutes, and re-suspended in 10 ml hypotonic solution (KCl 3 g/L, HEPES 4.8 g/L, EGTA 0.2 g/L, NaOH 0.36 g/L, pH7.4). This suspension was incubated at 37°C for 50 minutes. Subsequently, 2 ml of fixative (methanol acetic acid 3:1) was added, the cells were collected by centrifugation at 2000 RPM for 5 minutes and the pellet was re-suspended in 12 ml fixative. The cells were stored at -80°C until use. The SKy was performed by the SKy/FISH facility at the Roswell Park Cancer Institute (Buffalo, NY).

Whole chromosome painting was performed on metaphase chromosomes prepared as described above. Chromosome spreads were prepared as previously described [54]. Chromosome paints for chromosome 6 (starfish paints) were purchased from Cambio Ltd. (Cambs, UK). Hybridizations were performed according to the manufacturer's protocol. Fluorescent images were collected on a Leica TCS SP2 confocal microscope and edited using Velocity software.

Estimation of the maximum number of trisomic cells in a population was obtained from the following relationship: (1-f)^x ^= 1-p, where p is the probability of seeing at least 1 trisomic cell, x is the number of cells examined. and f is the frequency of trisomy in the population. When p is set at 0.95, and f at 0.05, X is 59. Thus, if one examines 59 cells and does not see a trisomic cell one can conclude with 95% confidence that the frequency of trisomic cells in the population is 5% or less.

## Authors' contributions

JSL derived MEF cell lines, maintained all cell lines (MEF & ES), mutagenized ES cells, performed FACS and analyzed FACS data, cloned variant ES cells, performed FISH and other fluorescent microscopy techniques, and analyzed cells for allele loss. JSL also contributed to manuscript preparation. MY performed timed mating of mice, and harvested and cultured blastocysts for the establishment of ES cell lines. JMF assisted in the establishment of ES cell lines and carried out much of the microsatellite analysis. SLS assisted in the establishment of ES cell lines and provided advice on experimental design and interpretation of data. JRS was the principal investigator and corresponding author. JRS conceived the project, helped with interpretation of data, contributed to authorship and final drafting of the manuscript. All authors read and approved the final manuscript.

## Figures and Tables

**Table 1:** Mutations produced by EMS in monochromatic ES cell clones.

**Monochromatic clone**	**Phenotype**	**Gene mutated**	**Mutation**	**Predicted protein alteration**
R26CY 1025 C-8	cyan	YFP	1 bp deletion	Truncated after 4^th ^amino acid
R26CY 1013 Y-1	yellow	CFP	G to A	G94D
R26CY 1025 Y-1	yellow	CFP	G to T	W60C

## References

[B1] Scrable HJ, Witte DP, Lampkin BC, Cavenee WK (1987). Chromosomal localization of the human rhabdomyosarcoma locus by mitotic recombination mapping. Nature.

[B2] Cavenee WK, Dryja TP, Phillips RA, Benedict WF, Godbout R, Gallie BL, Murphree AL, Strong LC, White RL (1983). Expression of recessive alleles by chromosomal mechanisms in retinoblastoma. Nature.

[B3] Cavenee WK (2003). The recessive nature of dominance. Genes Chromosomes Cancer.

[B4] Knudson AG (2002). Cancer genetics. Am J Med Genet.

[B5] Lefebvre L, Dionne N, Karaskova J, Squire JA, Nagy A (2001). Selection for transgene homozygosity in embryonic stem cells results in extensive loss of heterozygosity. Nat Genet.

[B6] Thiagalingam S, Foy RL, Cheng KH, Lee HJ, Thiagalingam A, Ponte JF (2002). Loss of heterozygosity as a predictor to map tumor suppressor genes in cancer: molecular basis of its occurrence. Curr Opin Oncol.

[B7] Engel E (1980). A new genetic concept: uniparental disomy and its potential effect, isodisomy. Am J Med Genet.

[B8] Pop R, Zaragoza MV, Gaudette M, Dohrmann U, Scherer G (2005). A homozygous nonsense mutation in SOX9 in the dominant disorder campomelic dysplasia: a case of mitotic gene conversion. Hum Genet.

[B9] Lasko D, Cavenee W, Nordenskjold M (1991). Loss of constitutional heterozygosity in human cancer. Annu Rev Genet.

[B10] Kaushal D, Contos JJ, Treuner K, Yang AH, Kingsbury MA, Rehen SK, McConnell MJ, Okabe M, Barlow C, Chun J (2003). Alteration of gene expression by chromosome loss in the postnatal mouse brain. J Neurosci.

[B11] Stern C, Rentschler V (1936). The Effect of Temperature on the Frequency of Somatic Crossing-Over in Drosophila Melanogaster. Proc Natl Acad Sci U S A.

[B12] Srinivas S, Watanabe T, Lin CS, William CM, Tanabe Y, Jessell TM, Costantini F (2001). Cre reporter strains produced by targeted insertion of EYFP and ECFP into the ROSA26 locus. BMC Dev Biol.

[B13] Liu X, Wu H, Loring J, Hormuzdi S, Disteche CM, Bornstein P, Jaenisch R (1997). Trisomy eight in ES cells is a common potential problem in gene targeting and interferes with germ line transmission. Dev Dyn.

[B14] Longo L, Bygrave A, Grosveld FG, Pandolfi PP (1997). The chromosome make-up of mouse embryonic stem cells is predictive of somatic and germ cell chimaerism. Transgenic Res.

[B15] Friedberg EC, Meira LB (2006). Database of mouse strains carrying targeted mutations in genes affecting biological responses to DNA damage Version 7. DNA Repair (Amst).

[B16] Mortensen RM, Conner DA, Chao S, Geisterfer-Lowrance AA, Seidman JG (1992). Production of homozygous mutant ES cells with a single targeting construct. Mol Cell Biol.

[B17] Cervantes RB, Stringer JR, Shao C, Tischfield JA, Stambrook PJ (2002). Embryonic stem cells and somatic cells differ in mutation frequency and type. Proc Natl Acad Sci U S A.

[B18] Munroe RJ, Bergstrom RA, Zheng QY, Libby B, Smith R, John SW, Schimenti KJ, Browning VL, Schimenti JC (2000). Mouse mutants from chemically mutagenized embryonic stem cells. Nat Genet.

[B19] Hawley TS, Telford WG, Hawley RG (2001). "Rainbow" reporters for multispectral marking and lineage analysis of hematopoietic stem cells. Stem Cells.

[B20] Elowitz MB, Levine AJ, Siggia ED, Swain PS (2002). Stochastic gene expression in a single cell. Science.

[B21] Raser JM, O'Shea EK (2004). Control of stochasticity in eukaryotic gene expression. Science.

[B22] Liu HS, Jan MS, Chou CK, Chen PH, Ke NJ (1999). Is green fluorescent protein toxic to the living cells?. Biochem Biophys Res Commun.

[B23] Zhao WN, Meng GL, Xue YF (2003). Labeling of three different mouse ES cell lines with the green fluorescent protein. Yi Chuan Xue Bao.

[B24] Kato M, Yamanouchi K, Ikawa M, Okabe M, Naito K, Tojo H (1999). Efficient selection of transgenic mouse embryos using EGFP as a marker gene. Mol Reprod Dev.

[B25] Ikawa M, Yamada S, Nakanishi T, Okabe M (1999). Green fluorescent protein (GFP) as a vital marker in mammals. Curr Top Dev Biol.

[B26] Ikawa M, Yamada S, Nakanishi T, Okabe M (1998). 'Green mice' and their potential usage in biological research. FEBS Lett.

[B27] Hadjantonakis AK, Dickinson ME, Fraser SE, Papaioannou VE (2003). Technicolour transgenics: imaging tools for functional genomics in the mouse. Nat Rev Genet.

[B28] Klysik J, Singer JD (2005). Mice with the enhanced green fluorescent protein gene knocked in to chromosome 11 exhibit normal transmission ratios. Biochem Genet.

[B29] Devgan V, Rao MR, Seshagiri PB (2004). Impact of embryonic expression of enhanced green fluorescent protein on early mouse development. Biochem Biophys Res Commun.

[B30] Hayes W (1968). The genetics of bacteria and their viruses; studies in basic genetics and molecular biology.

[B31] Eisenstein BI (1981). Phase variation of type 1 fimbriae in Escherichia coli is under transcriptional control. Science.

[B32] Yusa K, Horie K, Kondoh G, Kouno M, Maeda Y, Kinoshita T, Takeda J (2004). Genome-wide phenotype analysis in ES cells by regulated disruption of Bloom's syndrome gene. Nature.

[B33] Chan MF, van Amerongen R, Nijjar T, Cuppen E, Jones PA, Laird PW (2001). Reduced rates of gene loss, gene silencing, and gene mutation in Dnmt1-deficient embryonic stem cells. Mol Cell Biol.

[B34] Luo G, Santoro IM, McDaniel LD, Nishijima I, Mills M, Youssoufian H, Vogel H, Schultz RA, Bradley A (2000). Cancer predisposition caused by elevated mitotic recombination in Bloom mice. Nat Genet.

[B35] Borgdorff V, van Hees-Stuivenberg S, Meijers CM, de Wind N (2005). Spontaneous and mutagen-induced loss of DNA mismatch repair in Msh2-heterozygous mammalian cells. Mutat Res.

[B36] Sugawara A, Goto K, Sotomaru Y, Sofuni T, Ito T (2006). Current status of chromosomal abnormalities in mouse embryonic stem cell lines used in Japan. Comp Med.

[B37] Shao C, Deng L, Henegariu O, Liang L, Stambrook PJ, Tischfield JA (2000). Chromosome instability contributes to loss of heterozygosity in mice lacking p53. Proc Natl Acad Sci U S A.

[B38] Donahue SL, Lin Q, Cao S, Ruley HE (2006). Carcinogens induce genome-wide loss of heterozygosity in normal stem cells without persistent chromosomal instability. Proc Natl Acad Sci U S A.

[B39] Brown DG, Willington MA, Findlay I, Muggleton-Harris AL (1992). Criteria that optimize the potential of murine embryonic stem cells for in vitro and in vivo developmental studies. In Vitro Cell Dev Biol.

[B40] Burns EM, Christopoulou L, Corish P, Tyler-Smith C (1999). Quantitative measurement of mammalian chromosome mitotic loss rates using the green fluorescent protein. J Cell Sci.

[B41] Gross JB, Hanken J, Oglesby E, Marsh-Armstrong N (2006). Use of a ROSA26:GFP transgenic line for long-term Xenopus fate-mapping studies. J Anat.

[B42] Kalin TV, Wang IC, Ackerson TJ, Major ML, Detrisac CJ, Kalinichenko VV, Lyubimov A, Costa RH (2006). Increased levels of the FoxM1 transcription factor accelerate development and progression of prostate carcinomas in both TRAMP and LADY transgenic mice. Cancer Res.

[B43] Kim JE, Nakashima K, de Crombrugghe B (2004). Transgenic mice expressing a ligand-inducible cre recombinase in osteoblasts and odontoblasts: a new tool to examine physiology and disease of postnatal bone and tooth. Am J Pathol.

[B44] Kusakabe T, Kawaguchi A, Kawaguchi R, Feigenbaum L, Kimura S (2004). Thyrocyte-specific expression of Cre recombinase in transgenic mice. Genesis.

[B45] Chung SS, Cuzin F, Rassoulzadegan M, Wolgemuth DJ (2004). Primary spermatocyte-specific Cre recombinase activity in transgenic mice. Transgenic Res.

[B46] Cooley BC (2004). Murine model of neointimal formation and stenosis in vein grafts. Arterioscler Thromb Vasc Biol.

[B47] Kalinichenko VV, Gusarova GA, Tan Y, Wang IC, Major ML, Wang X, Yoder HM, Costa RH (2003). Ubiquitous expression of the forkhead box M1B transgene accelerates proliferation of distinct pulmonary cell types following lung injury. J Biol Chem.

[B48] Kohlhepp RL, Hegge LF, Nett JE, Moser AR (2000). ROSA26 mice carry a modifier of Min-induced mammary and intestinal tumor development. Mamm Genome.

[B49] Narita N, Bielinska M, Wilson DB (1997). Cardiomyocyte differentiation by GATA-4-deficient embryonic stem cells. Development.

[B50] Bex A, Vooijs M, Horenblas S, Berns A (2002). Controlling gene expression in the urothelium using transgenic mice with inducible bladder specific Cre-lox recombination. J Urol.

[B51] Nagy A (2003). Manipulating the mouse embryo : a laboratory manual.

[B52] [www.informatics.jax.org] MGI

[B53] [www.polymorphicdna.com] PDNAT

[B54] Henegariu O, Heerema NA, Lowe Wright L, Bray-Ward P, Ward DC, Vance GH (2001). Improvements in cytogenetic slide preparation: controlled chromosome spreading, chemical aging and gradual denaturing. Cytometry.

